# *SMN1 *dosage analysis in spinal muscular atrophy from India

**DOI:** 10.1186/1471-2350-6-22

**Published:** 2005-05-23

**Authors:** Akanchha Kesari, Hanna Rennert, Debra GB Leonard, Balraj Mittal

**Affiliations:** 1 Department of Genetics, Sanjay Gandhi Postgraduate Institute of Medical Sciences, Lucknow-14, U.P, India; 2 Department of Pathology and Laboratory Medicine, University of Pennsylvania, Philadelphia USA; 3 Current Address- Center for Genetic Medicine, Children's National Medical Center Washington- DC. USA; 4 Department of Pathology and Laboratory Medicine, Newyork Presbyterian Hospital, Cornell Campus, Newyork USA

## Abstract

**Background:**

Spinal muscular atrophy (SMA) represents the second most common fatal autosomal recessive disorder after cystic fibrosis. Due to the high carrier frequency, the burden of this genetic disorder is very heavy in developing countries like India. As there is no cure or effective treatment, genetic counseling becomes very important in disease management. *SMN1 *dosage analysis results can be utilized for identifying carriers before offering prenatal diagnosis in the context of genetic counseling.

**Methods:**

In the present study we analyzed the carrier status of parents and sibs of proven SMA patients. In addition, *SMN1 *copy number was determined in suspected SMA patients and parents of children with a clinical diagnosis of SMA.

**Results:**

wenty nine DNA samples were analyzed by quantitative PCR to determine the number of *SMN1 *gene copies present, and 17 of these were found to have one *SMN1 *gene copy. The parents of confirmed SMA patients were found to be obligate carriers of the disease. Dosage analysis was useful in ruling out clinical suspicion of SMA in four patients. In a family with history of a deceased floppy infant and two abortions, both parents were found to be carriers of SMA and prenatal diagnosis could be offered in future pregnancies.

**Conclusion:**

*SMN1 *copy number analysis is an important parameter for identification of couples at risk for having a child affected with SMA and reduces unwarranted prenatal diagnosis for SMA. The dosage analysis is also useful for the counseling of clinically suspected SMA with a negative diagnostic SMA test.

## Background

With a prevalence of 1 in 10,000 live births and a carrier frequency of approximately 1 in 50 [1], proximal spinal muscular atrophy (SMA) represents the second most common fatal autosomal recessive disorder after cystic fibrosis [2]. SMA is characterized by the degeneration of anterior horn cells of the spinal cord, resulting in progressive weakness. The condition is clinically heterogeneous and has been divided into four subtypes according to age of onset and clinical severity [3]. Molecular genetic analysis has mapped all four forms of childhood and adult SMA to chromosome 5q11.2-q13.3, suggesting that they are allelic disorders.

In a majority of normal individuals in the population, survival motor neuron *(SMN) *genes are present in at least one telomeric *(SMN1) *and one centromeric *(SMN2) *copy per chromosome. However, 26% of all normal chromosomes 5 lack *SMN2 *copy of the gene. The two *SMN *genes are highly homologous but a single nucleotide variation in exon 7 of *SMN1 *and *SMN2 *genes is responsible for functional differences [4]. The majority of SMA patients, irrespective of their clinical types, have homozygous deletion of the *SMN1 *gene [5]. In addition intragenic mutations have been identified in most of the patients who have only one copy of *SMN1 *gene, confirming the involvement of *SMN1 *in the pathogenesis of SMA [6]. Normal individuals with one copy of the *SMN1 *gene are carriers for this autosomal recessive disorder.

The single nucleotide differences in exons 7 and 8 are used to distinguish *SMN1 *and *SMN2 *in diagnostic and prenatal testing for SMA [7,8]. Although this methodology can detect homozygous absence of *SMN1*, it cannot differentiate the presence of one copy from two or more copies of *SMN1*. In recent years, molecular diagnostic testing for *SMN1 *copy number by dosage analysis has been developed [9,10], and is used to determine the carrier status for SMA in the majority of cases. However, in 3.7% of carriers, referred as "2+0" carriers, two copies of the *SMN1 *gene are present on one chromosome 5 and a deletion of *SMN1 *allele is present on the other chromosome 5 [11]. This situation cannot be distinguished by dosage analysis alone. In such cases dosage analysis in combination with linkage analysis for extended family members may be required to unequivocally determine whether an individual is "2+0" (a carrier) or "1+1" (not a carrier) [10].

Due to high carrier frequency, the burden of this genetic disorder is very heavy and genetic counseling is an active component in the disease management. In many cases the index patient dies without proper clinical diagnosis due to limited facilities and lack of awareness in primary care providers in India [12]. Therefore, carrier testing is particularly useful to direct genetic counseling for prenatal diagnosis. This is the first study from India using testing for the carrier status of parents and sibs of patients with a proven diagnosis of SMA. Additionally, SMA carrier testing has been performed for suspected SMA patients and parents of children clinically suspected to have SMA.

## Methods

### Subjects

Detailed clinical history and pedigree was drawn for all the cases and their families. Clinical examinations of patients (if alive) and family members were carried out in the Genetics Outpatient Department at Sanjay Gandhi Post Graduate Institute of Medical Sciences. Informed consents were obtained from all study members following genetic counseling. Twenty-nine individuals included in the study were divided into three categories. Group I comprised 14 parents and sibs of *SMN1 *gene deletion positive cases. Group II comprised 10 individuals, four couples, and two sibs whose deceased child (or children) or sibs were suspected to have SMA. Group III comprised 5 patients with a strong clinical suspicion of SMA but did not show homozygous deletions of exons 7 and 8 of the *SMN1 *gene. Five ml blood was collected in EDTA tubes from all individuals enrolled in the study.

### DNA isolation and PCR

DNA was extracted from blood samples using DNA extraction kit (Qiagen, Germany) according to the manufacturer's instructions. Patients were tested for homozygous deletion of exons 7 and 8 of *SMN1 *gene by a Polymerase Chain Reaction-Restriction Fragment Length Polymorphism (PCR-RFLP) method as described previously [13].

#### *SMN1 *copy number assay

The SMA carrier test determines the *SMN1 *gene copy number by comparing the levels of amplified products generated from exon 7 of *SMN1 *and a two-copy control gene following PCR amplification. Multiplex PCR of exon 7 *SMN1 *genomic sequences, *CFTR *exon 4 genomic sequences, and modified *SMN1 *and *CFTR *internal standard (IS) was performed using hexachloroflorescein labeled primers. Primer sequences and PCR amplification conditions were according to McAndrew et al (1997) [9] and Chen et al (1999) [10]. Briefly, PCR was performed in a 50 μl reaction mix containing 10 mM Tris-HCl (pH-8.3) PCR buffer, 1.5 mM MgCl_2_, 50 mM KCl, 0.01% (w/v) gelatin (Applied BioSystems Inc., (ABI) CA, USA), 1 mM spermidine, 0.05 mM each primer, 400 mM each dNTP, 1 unit Ampli Taq Polymerase (ABI) and 200 ng of genomic DNA. Internal Standards at approximately equimolar concentration to the genomic templates (6.4 × 10^4 ^copies) were added to each reaction tube. PCR amplification conditions were as follows: initial denaturation at 95°C for 5 min followed by 22 cycles of denaturation for 30 sec at 95°C, annealing for 30 s at 55°C, and elongation for 2 min at 72°C, followed by a final extension step for 10 min at 72°C. Each patient sample was analyzed in duplicate, and controls included 5 known two-copy, a one-copy and a homozygous deleted DNA samples, as well as water (no DNA template control).

After PCR, 4 μl of amplified DNA from each reaction was digested in a 6 μl reaction volume with 1 unit of Dra I (New England Biolabs, USA) for 3 hours at 37°C. Two μl of the Dra I digestion were mixed with 4 μl of formamide, 0.5 μl of Gene Scan-500 Rox (ABI) and 0.5 μl of loading buffer, heated at 95°C for 5 min and then placed on ice for 2 min. Digested PCR product mixtures were analyzed on an ABI 373 Genetic Analyzer instrument (ABI). Peak areas of the various products were determined using ABI Gene Scan 672 Software (ABI).

The *SMN1 *copy number was calculated using peak areas as follows: (*SMN1 *genomic/*SMN1*-IS) / (*CFTR *genomic/ *CFTR*-IS). Ratios were then normalized to the mean of five control samples with two copies of *SMN1 *gene.

## Results

**Figure 1:**
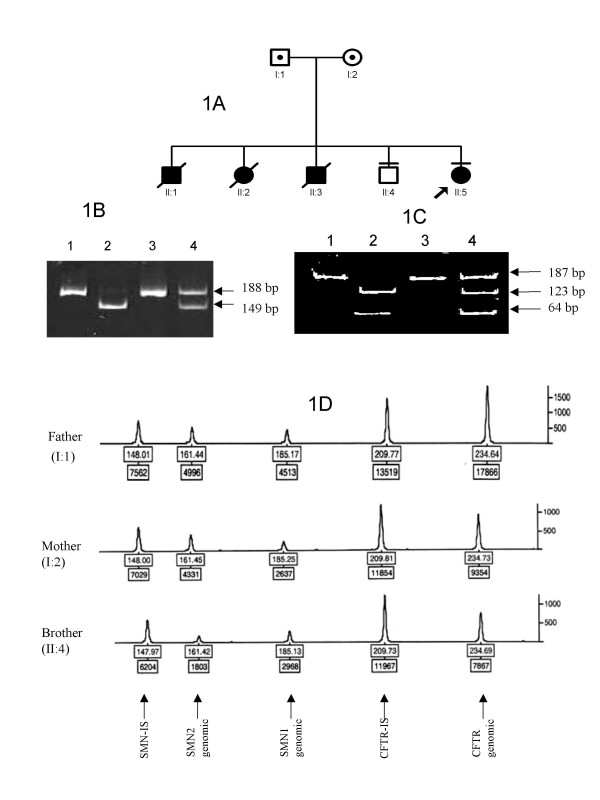
*SMN1 *copy number analysis for the family of Case 3. **1A **The pedigree of the family of Case 3. **1B: **Exon 7 PCR-RFLP Polyacrylamide gels showing the undigested and digested products of *SMN *exon 7 after *Dra *I digestion. Lane 1, undigested product of patient; Lane 2, digested product of patient with *SMN1 *deletion; Lane 3, undigested product of sibling; Lane 4, digested product of sibling with no *SMN1 *deletion. **1C: **Exon 8 PCR-RFLP after *Dde *I digestion. Lanes 1 and 3, undigested PCR product; Lane 2, digested product of patient with homozygous deletion of exon 8 of the *SMN1 *gene; Lane 4, digested product of sibling with no homozygous deletion of *SMN1*. **1D **Quantitative PCR gel scans of Father (I: 1), Mother (I: 2) and sibling (II: 4). Band sizes in base pairs are shown in the upper boxes and peak areas in the lower boxes. IS, internal standards.

Fig 1 shows the pedigree for Case 3 (Fig 1A), results of PCR-RFLP for exons 7 (Fig 1B) and 8 (Fig 1C) of *SMN1 *gene, and *SMN1 *dosage analysis gel scans (Fig 1D). The index patient had homozygous deletion of both exons 7 and 8 of *SMN1 *gene as shown by the absence of 188 bp and 187 bp bands, respectively (Fig. 1B and 1C, lanes 2 and 2, respectively). Dosage analysis gel scans of the father, mother, and sib are shown with band sizes (in base pairs) and peak areas under each peak (Fig 1D). The number of *SMN1 *gene copies was calculated as described in Methods. Normalized results (the mean of five control samples with two copies of the *SMN1 *gene) were consistently within the ranges of 0.8–1.2 for normal controls with two copies of *SMN1 *gene.

Fourteen samples in Group I (Cases 1–6) were from the parents and sibs of SMA patients with homozygous deletion of both exons 7 and 8 of *SMN1 *gene. Out of these, all parents and three of the four sibs had one *SMN1 *gene copy and one sib had two *SMN1 *gene copies (Table 1). In Group II (Cases 7–10), carrier analysis was performed on the basis of family history of SMA. In this group, five parents had two *SMN1 *copies (Cases 7, 8, 9) and three parents had one *SMN1 *gene copy (Case 9 and 10). Case 10 had two children and they showed two copies of *SMN1 *gene, respectively (Table 1). Five samples in Group III were analyzed to determine the copy number of *SMN1 *gene as these patients had strong clinical features consistent with SMA but did not have homozygous deletion of exons 7 and 8 of the *SMN1 *gene. Out of these, only a single patient had one copy of *SMN1 *gene, and the remaining four cases had two copies of *SMN1 *gene (Data not shown).

## Discussion

Twenty-nine samples were analyzed by quantitative PCR to determine the number of *SMN1 *gene copies present, and 17 of these were found to have one *SMN1 *gene copy. In the previous study, it was reported that 94.3% of normal individuals had two *SMN1 *copies and 2.1%, 0.7% and 2.9% had three, four and one copy, respectively [14]. Only one *SMN1 *gene copy is sufficient for normal functioning in an individual, as all parents with one copy of the *SMN1 *gene are asymptomatic.

From our small group of SMA cases, parents of confirmed SMA patients were obligate carriers of the disease and this was confirmed by SMA carrier testing. However, parents of children with SMA may not always be carriers as *de-novo *deletions of the *SMN1 *gene occurs in more than 2% of patients, with SMA [10,15]. Presence of *de-novo *deletion in the family lowers the recurrence risk for the couples from 25% to the risk of a second *de-novo *mutation which is very low. Knowledge of the carrier status of parents of affected children is useful for determining if a *de-novo *mutation has occurred and establishing the couple's future risk of having an affected child. If the parents are found to be carriers, then carrier testing can be offered to the siblings of the parents, who have a 50% risk of also being a carrier for SMA [1].

It has been reported that a small proportion of parents may have a "2+0" genotype in which there are two *SMN1 *gene copies on one chromosome and none on the other. In such cases a normal dosage analysis should be followed by linkage analysis of the family in order to try to distinguish between individuals who carry one *SMN1 *gene on each chromosome and those with a two-copy allele [1]. The recurrence risk for the family in which the "2+0" genotype is present is 25%.

Copy number analysis is also useful for testing of patients with a clinical diagnosis of SMA who are negative by a SMA diagnostic test that looks for homozygous deletion of exon 7 and 8 of the *SMN1 *gene. SMA is one of a wide spectrum of muscle and nerve disorders such as Becker muscular dystrophy, myotonic dystrophy, and Charcot-Marie tooth disease (Type IA) [16] that affect infants and young children. Clinical symptoms among these disorders are overlapping and may not be sufficiently specific to make a reliable clinical diagnosis of SMA. Patients that are referred for molecular genetic testing generally have symptoms, like hypotonia, floppiness, proximal muscle weakness, and loss of ambulation. These symptoms are not specific to 5q-linked SMA and in infants there are additional complications. In such circumstances molecular genetic testing for SMA is the best method to confirm the clinical diagnosis [17,18]. Muscle testing (EMG examination) at times may be difficult to perform in a neonate.

Homozygous deletion of the *SMN1 *gene confirms the diagnosis of 5q-linked SMA. If no homozygous deletion is detected, then *SMN1 *copy number analysis may be used if there is a strong clinical suspicion of SMA, since there is a 1 in 50 carrier frequency in the general population. An individual with signs and symptoms suggestive of SMA who does not lack at least one copy of the *SMN1 *gene is less likely to have 5q-linked SMA. For example, in the Group III patients, four of five had two copies of *SMN1 *gene and therefore were unlikely to have 5q-linked SMA. In such situations, the possibility of related disorders other then 5q-linked SMA should be considered. However, in patients with one copy of the *SMN1 *gene, the chances of a non-deletion type of mutation in the other allele may be explored [1,6].

The most severe form of SMA occurs at birth or in early infancy and may be difficult for primary care providers in India to diagnose. No data are available on the population prevalence of SMA and the status of diagnosis of SMA from India, due to the limited number of centers and the high cost and complexity of the molecular genetic test. So in many cases the child usually expires before the diagnosis is confirmed and the parents approach with a history of a previous child's death with symptoms consistent with SMA. In absence of a sample for molecular genetic testing for SMA, the information obtained by *SMN1 *copy number analysis for the parents can be utilized to confirm the diagnosis for the deceased child and to offer prenatal diagnosis for future pregnancies. The presence of one copy of the *SMN1 *gene in the parents will confirm their carrier status and prenatal diagnosis can be clearly advised in subsequent pregnancies. The family in case 10 had a history of a deceased floppy infant and two abortions. No surviving affected child was available. After *SMN1 *copy number analysis, both parents were found to have one *SMN1 *copy of the gene. Prenatal diagnosis was offered to the family and the fetus was found to be normal. Another child was born in the family and he was normal and dosage analysis showed two copies of *SMN1 *gene.

If both parents have two or more copies of *SMN1 *gene, as for the parents of Cases 7 and 8, then prenatal diagnosis for SMA will not be useful for future pregnancies. However, if only one of the parents has one *SMN1 *copy, as was seen in Case 9, then additional testing could be performed to clarify whether the two-copy parent is 1+1 or 2+0 using carrier testing and linkage analysis of additional family members. If the two-copy parent is 2+0, then prenatal diagnosis using the diagnostic test is still useful. If the two-copy parent is 1+1, then the affected child will usually have one *SMN1 *copy, and dosage analysis can be used for prenatal diagnosis, with a two-copy result indicating the fetus will not be affected. If the fetus has one *SMN1 *copy, then linkage analysis can be used, if DNA is available from an affected child. If only one parent has one copy of *SMN1 *gene, the situation may be clearly discussed with the parents before any prenatal testing is considered.

## Conclusion

Our results confirm that *SMN1 *copy number analysis is an important parameter for identification of couples at risk for having a child affected with SMA and reduces unwarranted prenatal diagnosis for SMA. Copy number analysis is also useful in the setting of clinically suspected SMA with a negative diagnostic SMA test.

## Competing interests

The author(s) declare that they have no competing interests

## Authors' contributions

AK and HR carried out the molecular genetic studies, participated in analyzing the data & drafted the manuscript. DGBL helped in analyzing the data and gave valuable suggestions in preparing the manuscript. BM participated in the designing of the study & manuscript. All authors read and approved the final manuscript.

## Pre-publication history

The pre-publication history for this paper can be accessed here:



## Figures and Tables

**Table 1:** Copy number of *SMN1 *gene in parents, sibs and SMA patients

**Case number**	**Subjects**	**Copy number of *SMN1 *gene**
**Group I**		

**Case 1**	P	0
	F	1
	M	1
	S	1

**Case 2**	P	0
	F	1
	M	1

**Case 3**	P	0
	F	1
	M	1
	S	2

**Case 4**	P	0
	F	1
	M	1

**Case 5**	P	0
	F	1
	M	1

**Case 6**	P	0
	S1	1
	S2	1

**Group II**		

**Case 7**	F	2
	M	2

**Case 8**	F	2
	M	2

**Case 9**	F	1
	M	2

**Case 10**	F	1
	M	1
	S1	2
	S2	2

P: Proband; F: Father; M: Mother; S: Sibling
